# Case Report: Vertebral Artery Dissection After Use of Handheld Massage Gun

**DOI:** 10.5811/cpcem.2022.2.56046

**Published:** 2022-04-06

**Authors:** Kathryn Sulkowski, Georgina Grant, Thomas Brodie

**Affiliations:** * University of Nevada, Las Vegas, Emergency Medicine Residency Program, Las Vegas, Nevada; † Mike O’Callaghan Federal Medical Center, Department of Emergency Medicine, Las Vegas, Nevada

**Keywords:** vertebral artery dissection, handheld massage gun

## Abstract

**Introduction:**

Arterial dissection is well known as a potential cause of stroke in young patients. Vertebral artery dissection occurs most commonly in the setting of minor trauma but has been seen in cases of cervical manipulation. With advances in at-home therapeutic modalities for neck pain came the advent of handheld massage guns. These massage guns have gained considerable popularity in recent years, but their safety for use in the cervical region has not been well studied.

**Case report:**

In this case report, we discuss a 27-year-old female who presented with headache, neck pain, and dizziness who was found to have vertebral artery dissection after repetitive use of a handheld massage gun.

**Conclusion:**

In young patients presenting with headache, neck pain, and vague neurologic symptoms it is important to consider vertebral artery dissection as a cause of symptoms as it can lead to serious morbidity. When considering an inciting event such as minor trauma, it may also be important to assess whether there has been use of a handheld massage gun. Although causality is difficult to establish, with the increase in use of handheld massage guns we may find more frequent association between their use and vertebral artery dissection.

## INTRODUCTION

The reported incidence of vertebral artery dissection (VAD) is estimated to be 2.6–3/100,000.^1^ Vertebral artery dissection is a known cause of stroke in patients younger than 45 years of age. Unfortunately, because its clinical features and symptoms tend to be vague and/or nonspecific, diagnosis may not even be considered. Upon close review of the literature, we found very few cases reported of VAD secondary to neck massage and none related to the use of a handheld massage gun. Given the increase in popularity of at-home, handheld massage, the importance of safety while using these devices is of the utmost importance. In this report we describe a case of VAD in a young woman after the use of a handheld massage gun. She was treated with aspirin and discharged with no residual neurological deficits.

## CASE REPORT

A 27-year-old female presented to the emergency department (ED) with a two-week history of progressively worsening dizziness. She described the dizziness as a combination of vertiginous symptoms as well as disequilibrium. Over the prior four days she noticed a headache and neck pain. She denied any recent trauma but had recently begun using a handheld massage gun on her neck over the preceding three weeks. The patient denied any past medical history and used no medications regularly, except for occasional over-the-counter ibuprofen, which did not alleviate her symptoms. She had a family history significant for migraine headaches. She endorsed occasional alcohol and daily tobacco use but denied any illicit substance use. She denied fever, diplopia, blurry vision, photophobia, phonophobia, or vomiting but endorsed mild associated nausea.

Physical examination revealed a young female resting comfortably. Vital signs were all within normal limits. On examination, she demonstrated full range of motion of the neck without pain. She had no audible carotid bruit, no notable swelling, ecchymosis, or midline cervical spinal tenderness to palpation. On a detailed neurologic examination, the patient had a Glasgow Coma Scale of 15 and was alert and oriented to person, place, and time. She had a normal cranial nerve exam, full strength in the upper and lower extremities, normal reflexes, and no ataxia. She had a negative Romberg test and normal, rapid alternating movement testing and finger-to-nose testing.

Initial diagnostic evaluation demonstrated a normal complete blood count. Comprehensive metabolic panel demonstrated no abnormalities, and all electrolytes were within normal limits. Beta human chorionic gonadotropin was undetectable. She was administered 25 milligrams (mg) meclizine orally, 1000 mg acetaminophen intravenously, 10 mg prochlorperazine intravenously, and a one-liter bolus of lactated Ringer’s solution for her symptoms.

Because the patient had prolonged symptoms and endorsed a history of massage gun use, both a computed tomography (CT) without contrast of the head and a CT angiogram (CTA) with intravenous contrast of the head and neck were obtained. The CT of the head without contrast demonstrated no notable abnormalities. The CTA of the head and neck revealed a long segment of irregular stenosis of the right vertebral artery extending from the second to the fifth cervical vertebra, most compatible with a vertebral artery dissection (VAD).

The patient was administered 324 mg aspirin orally and was transferred and admitted overnight to a tertiary accepting center with vascular surgery capability. Vascular surgery was consulted and recommended non-operative management and suggested consultation with interventional radiology. Interventional radiology was consulted and recommended continuing aspirin, and admission for observation with neurological checks every four hours. The patient was neurologically stable during the admission and was discharged with prescriptions for 324 mg aspirin orally daily as well as 25 mg meclizine orally as needed. She was given follow-up with neuro-interventional radiology in two weeks as an outpatient. The patient did not re-present to our hospital for follow-up.

## DISCUSSION

Dissection of the vertebral arteries related to handheld massage devices is not well documented, and we found no case reports in the literature of handheld massage devices potentially leading to VAD. When considering the anatomy of the extracranial vertebral artery, it is susceptible to dissection in three segments: its origin at the subclavian artery; as it traverses the intervertebral foramen; or at the site of entry into the cranium.^2^ Neurologic sequelae of VAD vary widely based on the location of the dissection and the amount of ischemic damage to the posterior circulation territory (cerebellum, brainstem, and posterior cerebrum). Early identification of VAD is crucial to improving outcomes; thus, it is imperative to maintain a high index of suspicion.

**CPC-EM Capsule**

What do we already know about this clinical entity?

*Arterial dissection is a potential cause of stroke in young patients. Vertebral artery dissection (VAD) usually occurs with minor trauma but has been seen with cervical manipulation*.

What makes this presentation of disease reportable?

*We could find no prior reported cases of VAD associated with use of a handheld massage gun*.

What is the major learning point?

*In young patients presenting with headache, neck pain, and vague neurologic symptoms, it is important to consider VAD as a cause of symptoms*.

How might this improve emergency medicine practice?

*Given the rising popularity of handheld massage guns, emergencv physicians should be aware of the evaluation and management of VAD*.

Headache, neck pain, and dizziness are very common chief complaints that are evaluated in the ED and outpatient clinics. The clinician must make a distinction between patients who have benign conditions and patients with life-threatening conditions. Much of this determination rests upon clinical suspicion based on a patient’s history as well as the use of imaging modalities.

Handheld massage guns have risen in popularity and become more accessible to the everyday user. There are many new handheld massage guns on the market, ranging in price from $50 to over $1000 for higher end models.^4^ Most of these devices rely on percussive motion (low amplitude, high frequency) to relieve tension in muscles. On many models, pulses per minute can be adjusted. While most user instruction manuals caution against holding the device in one place or using the device on the neck, they do advertise its use on the posterior neck, trapezius, and shoulder muscles.

Unfortunately, despite the increase of popularity, proper use is not clearly demonstrated. In website image searches for these devices, many ads show models using the device around the upper and lateral neck as well as on more anterior muscle tissues. Users with either no background in medicine or knowledge of the underlying anatomy may not realize the significance of the vasculature beneath these tissues and how they may be damaged with the use of handheld massage guns. While the patient in our case did not suffer any long-lasting deficits, this may not always be the case. If handheld massage guns pose a risk when used in improper locations, consumers must be made aware of these potential consequences.

Management options for VAD are varied and based on numerous factors such as presentation, time of onset, and imaging results. Options include antiplatelet or anticoagulation medications, endovascular management, or vascular surgery.^3^ In patients with severe deficits, reperfusion therapy is an option to more immediately restore blood flow to areas of the brain that can be salvaged. These options include alteplase, tenecteplase, or mechanical thrombectomy. These therapies are not without risk as they have the potential to increase the size of the intramural hematoma. These therapeutic modalities have been studied much more thoroughly in cases of cervical artery dissections with minimal literature to support their use in cases of VAD.^5^,^6^,^7^

Anticoagulation or antiplatelet therapy is more widely used for VAD. 8,9 Medically stable patients, like our patient, can be started on low molecular weight heparin, direct oral anticoagulants or antiplatelet therapy based on the ABCD2 score, similar to the risk stratification performed on other transient ischemic attack/stroke patients. Those deemed low risk by the ABCD2 score are started on 324 mg of aspirin daily, whereas high-risk patients are placed on dual antiplatelet therapy with both aspirin and clopidogrel.^10^ Most cases of nontraumatic VAD are shown to heal within the first few months of the inciting event. In one study of patients with VAD, 62% of cases showed complete healing of the dissection at six months.^11^

## CONCLUSION

We report the case of a 27-year-old female who presented with two weeks of worsening dizziness and four days of neck pain who had recently used a handheld massage device and was subsequently found to have vertebral artery dissection. Although rare, VAD is a known cause of stroke, particularly in young patients. In this patient population with presenting symptoms of dizziness, headache, and/or neck pain it is important to consider VAD as early diagnosis is imperative in improving outcomes. It is also crucial to take a complete social history to determine whether there were any provoking factors, such as the use of a handheld massage device. Treatment with antiplatelet or anticoagulation may be appropriate in the correct patient population.

With the rise in popularity of handheld massage guns, more research must be performed in evaluating their safety. Although causality is difficult to establish, we may find an increase in incidence of VAD as popularity of these devices continues to rise. In some circumstances we may ultimately discover that the use of handheld massage guns may be implicated as a cause of VAD.

## References

[1] Finley A, Rogers B, Richards T (2015). Postpartum vertebral artery dissection. BMJ Case Rep.

[2] Dutta G, Jagetia A, Srivastava AK (2018). “Crick” in neck followed by massage led to stroke: uncommon case of vertebral artery dissection. World Neurosurg.

[3] Gomez-Rojas O, Hafeez A, Gandhi N (2020). Bilateral vertebral artery dissection: a case report with literature review. Case Rep Med.

[4] Perez O (2021). Premium massage guns in 2021: *Sports Illustrated* reviews.

[5] Zinkstok SM, Vergouwen MD, Engelter ST (2011). Safety and functional outcome of thrombolysis in dissection-related ischemic stroke: a meta-analysis of individual patient data. Stroke.

[6] Bernardo F, Nannoni S, Strambo D (2019). Intravenous thrombolysis in acute ischemic stroke due to intracranial artery dissection: a single-center case series and a review of literature. J Thromb Thrombolysis.

[7] Hoving JW, Marquering HA, Majoie CBLM (2017). Endovascular treatment in patients with carotid artery dissection and intracranial occlusion: a systematic review. Neuroradiology.

[8] Markus HS, Hayter E, CADISS trial investigators (2015). Antiplatelet treatment compared with anticoagulation treatment for cervical artery dissection (CADISS): a randomised trial. Lancet Neurol.

[9] Markus HS, Levi C, King A, Cervical Artery Dissection in Stroke Study (CADISS) investigators (2019). Antiplatelet therapy vs anticoagulation therapy in cervical artery dissection: the Cervical Artery Dissection in Stroke Study (CADISS) randomized clinical trial final results. JAMA Neurol.

[10] Johnston SC, Rothwell PM, Nguyen-Huynh MN (2007). Validation and refinement of scores to predict very early stroke risk after transient ischaemic attack. Lancet.

[11] Arauz A, Márquez JM, Artigas C (2010). Recanalization of vertebral artery dissection. Stroke.

